# The association between medical students' motivation with learning environment, perceived academic rank, and burnout

**DOI:** 10.5116/ijme.5ff9.bf5c

**Published:** 2021-01-28

**Authors:** Ronen Zalts, Nathaniel Green, Sean Tackett, Robert Lubin

**Affiliations:** 1 Rambam Health Care Campus, Haifa, Israel; 2 Technion American Medical Students Program, Bruce Rappaport Faculty of Medicine, Haifa, Israel; 3 Johns Hopkins Bayview Medical Center, Baltimore, Maryland USA

**Keywords:** Learning environment, medical students, extrinsic motivation, intrinsic motivation, burnout

## Abstract

**Objectives:**

To assess the correlations between extrinsic and intrinsic motivation, personal growth and quality of life with learning environment perceptions, perceived academic rank and burnout among medical students.

**Methods:**

Cross-sectional questionnaires were administered to medical students at three medical schools in Israel, Malaysia, and China, at the end of one academic year. Surveys included demographic data, students' perceived academic rank, two learning environment perceptions scales, and scales for personal growth, goal orientation, burnout and quality of life. Comparative analyses were made to determine the significance of relationships between the outcome measures and control variables, using a series of t-tests. Pearson correlation coefficients were used to test the hypothesis.

**Results:**

Sixty-four percent (400/622) of the students responded. Significant correlations were found between: intrinsic motivation (r_(398) _=.37, p<.001); personal growth (r_(398)_=.62, p<.001); and quality of life (r_(398)_= .48, p <.001) with higher learning environment perceptions, intrinsic motivation (r_(398)_= .21, p<.001); personal growth (r_(398)_ =.21, p< .001); and quality of life (r_(398)_=.18, p<.001) with perceived academic rank, and negative correlation between personal growth (r_(398)_ =-.38, p<.001); and quality of life (r_(398)_ =-.42, p<.001) with burnout.

**Conclusions:**

Intrinsic motivation, personal growth and quality of life are correlated with higher learning environment perceptions and perceived academic rank. Burnout is influenced by personal growth and quality of life. We suggest focusing on motivation profiles before acceptance to medical school and during studies.

## Introduction

Learning environment (LE) is an important factor in medical student well-being.^1^ It has been shown that students' well-being is a factor that might have an implication on burnout.^2^ Medical schools invest great expense and effort in selecting students that will flourish in their competitive academic environments and succeed in the challenging professional practice environments of the hospital and clinic.^3^^-^^5^ The LE is an important factor during the undergraduate period, and consists of the social interaction, organizational culture and structures and virtual spaces that surround and shape the learners' experiences, perceptions, and learning.^6^ Although medical students begin their studies with similar or better mental health than age-similar controls, it has been previously shown that medical students and physicians have higher rates of burnout compared with similarly aged college graduates pursuing other careers,^7^ or compared to the general U.S. population.^8^

Positive LEs are thought to enhance personal growth and quality of life.^9^ Personal growth was found to better explain the variance in LE than academic performance in a cohort of U.S. medical students.^10^ Students who reported positive personal growth at the end of the clerkship year and/or at the end of the pre-clinical phase, perceived the LE more favorably than did students who reported negative personal growth or no change in it. In other studies, favorable LE perceptions were associated with a better quality of life and less burnout.^11^^-^^13^

Motivation is an important factor that predicts better academic performance.^14^ Students with high extrinsic motivation are driven by grades, class rank and earnings, whereas students with high intrinsic motivation see learning and self-improvement as ends unto themselves. It has been shown that higher intrinsic motivation is associated with improved academic performance.^15^

The role of motivation in learning, especially among medical students, needs more investigation, mainly due to the difficulty of measuring motivation studies' outcomes and the theoretical framework of motivation that needs more clarity.

The aim of this study was to find the correlation between students' motivation profiles and the perception of the LE, academic rank and burnout, and if motivation profiles should be measured in medical education. Our hypothesis was that intrinsic and extrinsic motivation, personal growth and quality of life leads to higher perceptions of LE, higher academic rank, and less burnout.

## Methods

### Study design and participants

This was a cross-sectional study of medical students at the end of the 2013-2014 academic year. Three medical schools were participated: (1) Technion American Medical Student Program (TeAMS) – a 4-year graduate-entry program in Israel. Most students are from the U.S. or Canada; (2) Perdana University-Royal College of Surgeons in Ireland School of Medicine (PURCSI) – a 5-year school-leaver program in Malaysia, and (3) Peking Union Medical College (PUMC) – an 8-year program in Beijing, China. The data were collected as part of a larger cross-sectional survey with methods previously described.^13^ At TeAMS and PURCSI, students were in years 1-4 and at PUMC, students were in years 4-7, which represented similar stages of training.

This study has been reviewed and approved by each schools' local ethics committee, due to the fact that this is a questionnaire-based study, including receiving informed consent from all participants. The participants were enrolled on a voluntary basis. The questionnaires were answered non-anonymously, but the responses were de-identified and analyzed by a statistician who had no contact with students at any of the schools.

### Data collection method

Surveys included demographic data (age, gender, race, and year in medical school), students perceived academic rank (bottom, middle, or top third), and two scales measure learning environment perceptions, a personal growth scale, a goal orientation scale, and single items for burnout and quality of life.

#### Learning environment measures

1. The Johns Hopkins Learning Environment perceptions Scale (JHLES) includes 28 items, assessing students' perceptions of the medical school's LE in seven domains/distinct: (1) Community of Peers, (2) Faculty relationships, (3) Academic climate, (4) Meaningful engagement, (5) Mentorship, (6) Inclusion and safety, and (7) Physical space. The JHLES total scores range from 28 to 140. A high score indicates positive perceptions of the LE. Previous studies provide validity evidence for content, response process, internal structure, and relationship to other variables.^16^ JHLES has been applied in several medical schools in the USA, Malaysia, Taiwan, Israel, China, Brazil, and east India.^12^^,^^17^^-^^20^

2. The Dundee Ready Education Environment Measure (DREEM)^21^is the most widely used method to assess learning environments internationally. It includes 50 items grouped into five categories: (1) Perceptions of teachers, (2) Perceptions of teaching, (3) Academic self-perception, (4) Perceptions of atmosphere, and (5) Social self-perception. Each statement is ranked on a five-point scale, from 4 (strongly agree), to 0 (strongly disagree). A high score indicates positive perceptions of the LE.

#### Personal growth

The personal growth Scale assessed students' perceptions of the extent to which they were worse or better compared to when they started medical school. The scale was modified from a revised personal growth scale, using a 5-point Likert-type scale, from -2 (much worse) to +2 (much better).^22^^,^^23^ The sum of the seven items' ranks (range -14 to +14) indicating a decline in growth – negative scores, to an increase in growth – positive scores. A score of zero indicated no change. The scale's validity was established in a sample of residents: content validity, response process, internal structure and relationship with other variables.^16^

#### Goal orientation

The motivation was measured by three Patterns of Adaptive Learning Scales (PALS) measuring goal orientation.^24^ Two PALS scales, performance-approach Goal orientation and performance-avoid goal orientation measured extrinsic motivation, and one, mastery goal orientation, measured intrinsic motivation.^25^ The items are ranked on a 5-point, Likert-type scale from 1 (not at all true) through 3 (somewhat true) to 5 (very true).^26^ A high score indicates high motivation (extrinsic/intrinsic).

### Burnout

Burnout was measured by a two-item burnout scale. Respondents are requested to report how often they feel "burned out from my work" (emotional exhaustion) or "callous toward people" (depersonalization),^27^ along a 7-point Likert-type scale, from 1 (daily) to 7 (never). This scale was shown as a good abbreviated burnout assessment tool in medical students.^28^ A high score indicates low levels of burnout.

#### Quality of life

The quality of life was reported by one item. The respondents were requested to rate their overall quality of life on a 5-point Likert-type scale, from 1 (as bad as it can be) to 5 (as good as it can be). A high score indicates good quality of life.

### Data analysis

Gender, school, and student age were considered as potential control variables. School was used as a proxy for race.

Prior to conducting comparative analyses, three skewed scales were normalized using square root transformations. Comparisons were made to determine the significance of relationships between the outcome measures and potential control variables: differences between the three schools were assessed using analyses of variance with post hoc pairwise comparisons; gender differences were investigated using a series of t-tests; and Spearman correlations were conducted between student age and the outcome measures. Pearson correlation coefficients were computed between all study measures. Partial correlations were computed controlling for school, in order to avoid the confounding effect between the study variables and the medical school of the respondents. The differences between schools were examined prior to testing the hypothesis.^15^

## Results

A total of 622 questionnaires were administered to the students at the three medical schools. 400 students (TeAMS n=92, PURSCI n=160, and PUMC n=148) responded to the survey, with a total response rate of 64.3%. Demographic characteristics are presented in Table 1.

In the current study, a positive, high and significant correlation (r_(__398)_=.63, p<.001) was found between JHLES (M= 100.3, SD=15.2) and DREEM (M=118.3, SD= 28.3) indicating partial association between them (about 40% covariance).

### Association among outcome variables

The correlations between both measures of LE Perceptions (JHLES and DREEM) perceived academic rank were positive and low but significant (r_(__398) _=.13, p < .05); whereas their correlations with burnout were found negative, moderate and significant (JHLES r_(398)_=-.41, p<.001; DREEM r_(398)_=-.33, p< .001).

The correlation between perceived academic rank and burnout are negative and low but significant (r_(__398)_=-0.19, p< .001).

### Association among predictors

A positive, high and significant correlation was found between external motivation measures: performance-approach and performance-avoid (r_(__398)_=.57, p < .001). The correlations between the internal motivation – mastery measure and the two external motivation measures were very low.

A positive, moderate and significant correlation was found between internal motivation measure – mastery and personal growth (r_(__398)_=.32, p<.001) and quality of life (r_(398)_=.29, p< .001); whereas the correlations between external motivation measures: performance-approach (r_(398)_=.21,p<.001) and performance-avoid (r_(398)_=.08, p=.11) with personal growth are low.

The correlation between personal growth and quality of life are positive, moderate and significant (r_(__398)_=.49, p < .001).

### Intercorrelations between Predictors and Outcome Variables

Pearson correlations between predictors and outcome variables are presented in Table 2. As shown in the table, positive, moderate and significant correlations were found between intrinsic motivation (mastery goal orientation, r_(__398)_ =.37, p < .001), personal growth (r_(398)_= .62, p<.001), and quality of life (r_(398) _= .48, p<.001) – with higher LE perceptions, as measured by the JHLES. Similar associations were found for LE perceptions as measured by the DREEM. Low though significant correlations were found between the two measures of extrinsic motivation (performance-approach goal orientation r_(__398)_ =.15, p<.01 and performance-avoid goal orientation r_(398) _= .10, p < .05) – with higher LE perceptions (JHLES). The correlations with DREEM were found very low.

The correlations between personal growth (r_(__398) _=.21, p < .001), and quality of life (r_(398)_= .18, p < .001) – with perceived academic rank were found positive and low, but significant. Positive but low significant correlations were found between intrinsic motivation (mastery goal orientation, r_(__398) _= .21, p < .001), and extrinsic motivation (performance-approach goal orientation r_(398)_=.14, p<.01) with perceived academic rank. No correlation was found between extrinsic motivation (performance-avoid goal orientation, r_(__398) _= .07, p =.16) with perceived academic rank.

Negative, relatively low and significant correlations were found between intrinsic motivation (mastery goal orientation, r_(__398) _=-.12, p< .05), personal growth (r_(398)_=-.38, p< .001), and quality of life (r_(398)_ =-.42, p<.001) with burnout. Very low positive correlations were found between external motivation (performance-avoid goal orientation, r_(__398)_=.10, p<.05 and performance-approach goal orientation, r_(398)_=.05, p= .32) and burnout.

## Discussions

In this cross-sectional survey of medical students from three medical schools, our hypothesis was that positive correlations will be found between students' intrinsic and extrinsic motivation, personal growth and quality of life – and higher perceptions of LE and higher perceived academic rank. We found that increased intrinsic motivation had a moderate significant correlation with LE perceptions and perceived academic rank; Students with higher intrinsic motivation also reported slightly less burnout.

**Table 1:** Demographic characteristics of the students

Characteristics		Medical School				p-value
		TeAMS (n = 92)	PURSCI (n = 160)	PUMC (n = 148)	Total (n = 400)	
		n (%)	n (%)	n (%)	n (%)	
Response rate		92/121 (76)	160/198 (80.1)	148/303 (48.8)	400/622 (64.3)	
Gender						.24
	Male	38 (41.3)	59 (36.9)	66 (44.6)	163 (40.8)	
	Female	52 (56.5)	100 (62.5)	77 (52.0)	229 (57.2)	
	Missing	2 (2.2)	1 (0.6)	5 (3.4)	8 (2.0)	
Age (M ± SD)		26.1 ± 2.38	21.0 ± 0.95	22.8 ± 1.58	22.8 ± 2.45	
Race						<.0001
	White	69 (75.0)	0 (0.0)	0 (0.0)	69 (17.2)	
	Malay	0 (0.0)	41 (25.6)	0 (0.0)	41 (10.3)	
	Chinese	0 (0.0)	47 (29.4)	143 (96.6)	190 (47.5)	
	Indian	0 (0.0)	48 (30.0)	0 (0.0)	48 (12.0)	
	Other	9 (9.8)	11 (6.9)	0 (0.0)	20 (5.0)	
	Multiracial	3 (3.3)	4 (2.5)	0 (0.0)	7 (1.7)	
	Missing	11 (11.9)	9 (5.6)	5 (3.4)	25 (6.3)	
Marital Status						<.0001
	Single	60 (65.2)	160 (100)	141 (95.3)	361 (90.2)	
	Married	20 (21.7)	0 (0.0)	2 (1.3)	22 (5.5)	
	Divorced	1 (1.1)	0 (0.0)	0 (0.0)	1 (0.3)	
	Missing	11 (12.0)	0 (0.0)	5 (3.4)	16 (4.0)	
School Year						<.0001
	First	29 (31.5)	54 (33.8)	61 (41.2)	144 (36.0)	
	Second	19 (20.7)	52 (32.5)	20 (13.5)	91 (22.8)	
	Third	12 (13.0)	42 (26.2)	35 (23.7)	89 (22.3)	
	Fourth	21 (22.8)	0 (0.0)	32 (21.6)	53 (13.2)	
	Missing	11 (12.0)	12 (7.5)	0 (0.0)	23 (5.7)	
Future Intentions for Residency						<.0001
	Family Med.	5 (5.4)	9 (5.6)	2 (1.4)	16 (4.0)	
	Internal Med.	23 (25.0)	14 (8.8)	61 (41.2)	98 (24.5)	
	OB’/GYN	5 (5.4)	12 (7.5)	9 (6.1)	26 (6.5)	
	Pediatrics	20 (21.7)	24 (15.0)	0 (0.0)	44 (11.0)	
	Emergency Med.	3 (3.3)	9 (5.6)	1 (0.7)	13 (3.3)	
	General Surgery	6 (6.5)	28 (17.5)	38 (25.6)	72 (18.0)	
	Orthopedics	2 (2.2)	7 (4.4)	8 (5.4)	17 (4.2)	
	Other	18 (19.6)	45 (28.1)	29 (19.6)	92 (23.0)	
	Missing	10 (10.9)	12 (7.5)	0 (0.0)	22 (5.5)	

TeAMS, Technion American Medical Student Program, Haifa, Israel; PURSCI, Perdana University Royal College of Surgeons in Ireland, in Serdang, Malaysia; PUMC, Peking Union Medical College, Beijing, China

However, the correlation between extrinsic motivation measures and LE perceptions with perceived academic rank and burnout were found to be low. The association between extrinsic and intrinsic motivation and LE perceptions were previously studied, and the distinction between these two types of motivation may shed important light on both developmental and educational practices.^29^ It was shown that intrinsically-motivated students are more persistent in their studies.^30^ Specifically regarding medical students, positive correlation have been found between intrinsic motivation and healthy study habits, effort invested and, ultimately, performance.^15^

**Table 2:** Pearson(r) Intercorrelations between Predictors and Outcome Variables

		Motivation (goal orientation)			Personal Growth	Quality of Life
		Extrinsic Motivation		Mastery (Intrinsic Motivation)		
	Predictors^†^	Performance Approach	Performance Avoid			
Outcomes‡	M ± SD	3.1 ± 0.9	3.2 ± 0.9	4.4 ± 0.6	3.7 ± 0.7	3.5 ± 0.9
		r_(398)_	r_(398)_	r_(398)_	r_(398)_	r_(398)_
LE Perceptions (JHLES)	100.3 ±15.2	.15^**^	.10^*^	.37^***^	.62^***^	.48^***^
LE Perceptions (DREEM)^†^	118.3 ± 28.3	.11^*^	.05	.36^***^	.54^***^	.43^***^
Perceived Academic Rank	2.2 ± 0.7	.14^**^	.07	.21^***^	.21^***^	.18^***^
Burnout	3.2 ± 1.4	.05	.10^*^	-.12^**^	-.38^***^	-.42^***^

LE, Learning environment; M=Mean; SD=standard deviation; r=Pearson correlation coefficient. *p>=.05; **p>=.01; ***p>=.001. †Each predictor score ranges from 1 (worse) to 5 (best). ‡Correlations were partialled by school. See methods for value ranges of outcomes.

The major finding in the present study, is that intrinsic motivation had a stronger association with LE perceptions compared to extrinsic motivation measures. Previous studies that dealt with motivation among students and especially medical students revealed similar finding.^31^^-^^33^

The association of medical students' internal motivation with perceived academic rank was found positive and low, but significant, in contrast to extrinsic motivation. Motivation was not found to be associated with burnout, in contrast to previous studies (see for example Lyndon et al. 2017).^34^

Personal growth and quality of life were found to be strongly correlated with LE perceptions, less strong with perceived academic rank and negatively associated with burnout. The first two findings are consistent with previous findings.^35^

The findings on burnout are particularly interesting. Maslach postulated the importance of the community to protect against workplace burnout,^36^ leading to the development of interventions to improve peer relationships, such as social gatherings and "learning communities".^37^ Previous studies have shown the connection of burnout with unprofessional behavior; desire to quit medical school and suicidal ideation.^7^^,^^12^ As mentioned above, in this study, we found that motivation has a weak association with burnout, while personal growth and quality of life are protective resources. This supports the holistic approach to curriculum reform and further refinement of counseling to include a focus on developing intrinsic motivation.^38^ In order to improve medical students' achievements and outcomes, interventions should focus on achievement goal orientations, thus shifting from extrinsic motivation to intrinsic motivation. We found only one study which showed that extrinsic motivation can be formed into intrinsic motivation using rich technologies LE (use of computers in teaching, learning and assessment processes).^39^ The implications of this finding on selecting students to medical schools demands further investigation, as well as designing LE's that may enhance intrinsic motivation and diminished burnout.

Nevertheless, this study has some limitations. First, it was conducted on three academic settings, which increases generalizability, but also introduces new confounding variables. However, it is likely, considering the strength of association, that our findings would resist the diluting effect of the addition of further variables. Second, although many students participated, some degree of selection bias cannot be ruled out; it is possible, for example, that intrinsically motivated students would be more likely to participate in such a study. Third, all measures were self-reported, and do not necessarily reflect actual student behaviors or outcomes. Finally, and perhaps most importantly, cross-sectional study designs cannot prove causation.

## Conclusions

Our study provides evidence that intrinsic motivation is related to positive LE perceptions and is positively correlated with perceived academic rank. These characteristics are components of a student's well-being and may predict success in their career and life. This study supports that motivation profiles are an important component of a student's evaluation before acceptance to medical school and during studies. This should be considered as we develop programs to serve medical students and future physicians.

Necessary further studies should focus on the association between motivational profiles, ways to convert from extrinsic motivation to intrinsic motivation during medical education and the relationship between levels of intrinsic motivation and success in medical school. However, our study suggests that interventions aimed at selecting and reinforcing those who exemplify intrinsic motivation will yield benefits both for the individual medical student and improving learning environment perceptions.

### Acknowledgments

The authors gratefully acknowledge those who participated in initial data collection, Mrs. Linda Deacon for statistical analysis and Dr. Dalia R. Hasson-Gilad for revision and editing.

### Conflict of Interest

The authors declare that they have no conflict of interest.
